# Patient selection in genetic association studies in idiopathic scoliosis

**DOI:** 10.1186/1748-7161-8-S1-O2

**Published:** 2013-06-03

**Authors:** P Harasymczuk, P Janusz, T Kotwicki

**Affiliations:** 1 Department of Pediatric Orthopedics and Traumatology, Poznan, Poland

## Background

Although phenotypic presentation of scoliosis may be similar among cases, its genetic background may differ in relation to sex, age at presentation, curve type etc. It is difficult to compare results of genetic association studies published in the last 10 years because of different inclusion criteria and research approach [1].

## Aim

To analyze genetic association studies of IS, published up to date, with the aim of evaluating selection biases.

## Methods

We have evaluated available English language articles from the past ten years (2001-2011). Initial search was performed through PubMed, Medline, and Google Scholar with key words such as: idiopathic scoliosis, gene, genetic, SNP, association studies. As a result, more than 700 papers were obtained. Research method of each study was assessed. We included only genetic association studies or genome wide association studies. We ascertained 26 studies available for further analysis.

## Results

In total, the studies report on 11,797 patients and 23,867 controls tested. Out of those, 15 were performed on Chinese population, 4 studies on Japanese population, 4 studies on Caucasian population, 1 on Korean population and 2 on mixed or uncertain populations. 13 of the studies were performed on females only and 13 on males and females with known or unknown male to female ratio. One study did not report any IS inclusion criteria. 3 studies did not report any Cobb angle inclusion criteria, 11 included patients with Cobb angle below 20, 8 included patients with Cobb from 20 to 30, and 4 with Cobb angle over 30. Sixteen studies did not report curve types, and 10 had mixed type curve pattern.

## Conclusions

Most of the studies conducted up to date did not select cases on the basis of common inclusion criteria. This gives rise to risk of selection bias, population stratification and other biases rendering the results questionable. Common inclusion criteria for genetic association studies are needed.
